# Research ethics in inter- and multi-disciplinary teams: Differences in disciplinary interpretations

**DOI:** 10.1371/journal.pone.0225837

**Published:** 2019-11-27

**Authors:** Ambika Mathur, Sharon F. Lean, Caroline Maun, Natalie Walker, Annmarie Cano, Mary E. Wood

**Affiliations:** 1 Graduate School, University of Texas at San Antonio, San Antonio, Texas, United States of America; 2 Graduate School, Wayne State University, Detroit, Michigan, United States of America; 3 Department of English, Wayne State University, Detroit, Michigan, United States of America; 4 Office of the Provost, Wayne State University, Detroit, Michigan, United States of America; Universidade de Mogi das Cruzes, BRAZIL

## Abstract

As research teams are increasingly comprised of members from multiple disciplines, ranging from the physical sciences, life sciences, social and behavioral sciences to the arts and humanities, it is important to revisit how research is conducted at several levels. Coupled with the national concern over rigor and reproducibility in research, it is therefore crucial to ensure that all members of such multidisciplinary teams view the need for ethics in the conduct of research in similar ways. Towards this end, Wayne State University developed a course in the Responsible Conduct of Research (RCR) which was mandatory for all its 1500 doctoral students across all disciplines in its 75 PhD programs. We found that student perceptions of the validity, applicability and usefulness of the course varied by discipline. This was in spite of iterative changes made to the course by faculty in those disciplines to make the content palatable to all. The findings show that more work needs to be done to fully incorporate the needs of social sciences and humanities disciplines in a comprehensive university course. This is especially important as these students become members of large multidisciplinary research teams in order to uphold the highest levels of rigor, reproducibility and ethics.

## Introduction

Interdisciplinary research integrates teams with diverse perspectives and training to advance knowledge and solve challenging problems. The National Science Foundation, which has long promoted the value of interdisciplinary research, defines it as “…a mode of research by teams or individuals that integrates information, data, techniques, tools, perspectives, concepts, and/or theories from two or more disciplines or bodies of specialized knowledge to advance fundamental understanding or to solve problems whose solutions are beyond the scope of a single discipline or area of research practice.[1].” Increasingly, interdisciplinary approaches are embraced in academia as a means to go beyond the narrow focus of a single discipline to approach research questions in new and innovative ways.

Against this backdrop, instruction in responsible conduct of research (RCR) is an important practice that helps doctoral students understand accepted norms and standards of research and scholarship in every discipline [2]. As a best practice in graduate education, high-quality training can enhance a doctoral student’s skill set and foster a lifelong career based on integrity and rigorous ethics [3]. RCR instruction serves not only to enhance the research experience for students, but also increases the rigor and reproducibility of research. Public confidence in research findings can be seriously undermined when fraud or misconduct are brought to light, especially in high profile cases of plagiarism, conflict of interest, or fraudulent practices in data collection, analysis, and reporting [4,5]. While some cases of research misconduct are due to malicious intent, other cases may be due to a lack of training in ethical decision-making. Recklessness, while not as severe as research misconduct, also diminishes integrity in research [6]. Recklessness is defined as an indifference to truth, which can be manifested as sloppiness and carelessness. This can result in research documents that contain fabricated or false data. In such cases, the researcher should have known better, whether the oversight was intentional or not. Better management, collegial oversight, training and communication are identified as ways to minimize recklessness. Resnick [7] calls on faculty and mentors to clearly distinguish the differences between negligence and reckless practices when training the next generation of researchers.

This paper describes a comprehensive RCR training course developed by Wayne State University’s Graduate School, which embraces an interdisciplinary approach to serve the needs of all students to develop a deeper appreciation and understanding of ethics training. Classified as a "doctoral university: highest research activity" by the Carnegie Classification of Institutions of Higher Education, Wayne State has approximately 1500 PhD students across 75 doctoral programs. We describe the evolutionary process of designing, evaluating and modifying the training using a multidisciplinary approach to support students and engage faculty. We hope this model can be easily adapted by other universities that seek the efficiency and quality control of a centralized course. Another value of comprehensive, institution-wide RCR training is that it signals an institutional commitment to the value of integrity in research [8–10]. Our training is mandatory and required of all PhD students and highly recommended to postdoctoral fellows. With required and centralized training, we consistently communicate the importance of an ethical research climate with the goal of equipping our students with the knowledge and tools to adhere to the highest standards of research integrity.

Issues related to RCR are common in all fields, but their frequency and type vary by discipline. For example, falsification of data may be more prevalent in the sciences, but plagiarism is an issue for all disciplines, including the arts and humanities [11–13]. Conflicts of interest have been documented in the life sciences with industry-funded research [14]. On the other end of the disciplinary spectrum, ethical challenges have arisen in the social sciences.[15,16]. In particular, evaluating the risks of research involving human subjects is not always straightforward as it may be extremely hard to provide protections that adequately minimize risk to study participants [17]. Laitin and Reich, lamented the lack of a required RCR course in most political science departments, noting that "[p]rofessional-ethics courses are standard in business and law curricula, and it seems a mistake to assume that our students learn our ethical aspirations through osmosis or tangentially through methods courses [15]”.

RCR training serves another key purpose and refutes the notion that character is fixed in adults. The research literature argues that adult socialization is a key process in preparing doctoral students for careers in research and scholarship [18]. RCR training prepares students for these roles and inculcates them with the values of the profession [19]. Expectations are clearly conveyed during this socialization process resulting in increased collegiality and shared values and norms [20].

At the minimum, scholars should have a thorough understanding of academic integrity and ethical behavior in their own fields [10]. However, we argue that a basic level of exposure to RCR issues in a broad spectrum of fields has long-term value. The academic and non-academic workplace is undergoing transformation and multidisciplinary approaches are necessary in a rapidly changing labor market. It clearly behooves graduate educators to prepare their students to work in collaborative teams by exposing them to a multidisciplinary mindset.

The need to provide comprehensive training originally stems from mandates established by the U.S. Congress and federal funding agencies in the 1980s for biomedical and behavioral science research [21]. The mandate was expanded to include all federal agencies and federally funded research [10]. Furthermore, the accreditation guidelines of the Higher Learning Commission (HLC) require universities to develop policies and procedures to support research ethics and provide guidance to students in the ethical use of information [22].

While RCR training is necessary for federal compliance, little guidance was provided on implementing the mandates [21,23]. The result has been a widespread practice of limiting RCR training to students supported by federal grants by completion of an online course [24]. However, online-only resources fail to address ethical issues students will face in diverse research environments and workplaces [25]. A 2008 workshop funded by the National Science Foundation (NSF) at the National Academy of Engineering (NAE) identified criteria for robust RCR training. They include that training should be for all students; it should be delivered in multiple formats and not only through online modules; and that content should vary by discipline and career stage [23]. Furthermore, the literature suggests that on-line training is critical in introducing RCR concepts to participants to prepare them for the more interactive and discussion-based face-to-face training [26]. Providing on-line training not only lays a solid foundation for in-person training, but it also can reduce duplication in course content between the tutorials and face-to-face training. To address these best practices, Wayne State’s RCR program is delivered in four distinct stages and is required of all doctoral students based on the recognition that all research and scholarly work can be costly to society if it is ethically compromised [24]. In addition, course content has been tailored in more recent iterations to specific disciplines in accordance with the NSF/NAE criteria.

### Four stages of the RCR course

Participation in the RCR course is mandatory for all doctoral students and postdoctoral scholars in their first year, preferably in their first semester. They must register for and enroll in a zero-credit course allowing the Graduate School to document participation and completion of the requirements. Students who have not completed RCR training by the end of the first year in their program will have a registration hold until they demonstrate a plan to complete the course.

The course has four stages. Stage I involves completion of a specifically-developed Collaborative Institutional Training Initiative (CITI) online course Stage II is a one-day in-person Saturday workshop incorporating lectures and small group discussions. Stage III is the departmental/mentor-based training in specialized RCR topics specific to the student’s discipline. Stage IV requires students to write an essay describing what they have learned in the previous three stages and how they will employ this knowledge in their everyday work life. Stages I and II incorporate general RCR materials that are relevant for all disciplines. Stage III provides training specific to the student’s area of research. Stage IV allows students to integrate their RCR learning across the general and specific domains.

#### Stage I

Common RCR content is delivered through CITI online modules (see S2 File CITI Training). Students are required to complete the modules two weeks before attending the Stage II Saturday workshop. The online modules do not need to be completed in one sitting. Topics in the Stage I online component include core areas that apply to all PhD students regardless of discipline including: (a) introduction to RCR (b) authorship, (c) collaborative research, (d) conflict of interest, (e) data management, (f) financial responsibility, (g) mentoring, (h) peer review, (i) plagiarism, (j) research misconduct and (k) research ethics. A certificate of completion is issued to each student once they complete the online modules.

#### Stage II

Stage II features a full-day workshop held twice a year on a Saturday. Students must attend one of the two sessions in their first year. The morning session consists of several short faculty lectures on core RCR topics with reference to the WSU context and related 0university resources. Topics include: (a) mentoring and the individual development plan; (b) conflict resolution; (c) communication strategies; (d) data management/recordkeeping; (e) reporting research misconduct and whistleblower protection; (f) peer review; (g) authorship and plagiarism; and (h) conflicts of interest. Lectures are recorded so students can review materials later if they choose.

In the afternoon, faculty and postdoctoral scholars lead breakout groups in a discussion of four different case studies. The case studies are assigned to subgroups of four and all students must lead the discussion of one case. The composition of the subgroups is selected randomly to include students from different disciplines in order to promote interdisciplinary perspectives. Subgroups then reconvene with the full class and discussion leaders to address questions arising from the cases. A sample of case studies can be found in S3 File.

#### Stage III

Stage III training occurs in the student’s home department to ensure that discipline-specific instruction is provided by faculty. Departments vary greatly in their delivery, course content and methodology of the Stage III component. For example, this training may be provided as a credit-bearing course or part of a for-credit course; as a workshop or series of workshops; through one-on-one discussions between students and their faculty advisors; or brown bag meetings with faculty and students. Departments are strongly encouraged to tackle difficult or ambiguous topics with students to enhance knowledge of research ethics.

#### Stage IV

Once a student has completed Stages I-III, to finalize the course, they must submit online a short essay which responds to questions such as: (a) describe the knowledge you gained from Stages I and II of the course; (b) describe your departmental RCR training in Stage III; (c) describe how you will operationalize RCR knowledge in all aspects of your research. The essays are evaluated by RCR course faculty. After Stage IV is complete, a pass or fail grade is entered, which becomes part of the student transcript. Students who successfully complete the course also receive a link allowing them to request a research ethics micro-credential, which is a digital badge that can be shared on LinkedIn or other social media [27].

## Materials and methods

In fall 2016, a centralized RCR course was made mandatory for all new Ph.D. students and postdoctoral scholars because the principles, practices and ethics mandated by federal funding agencies apply broadly to all students conducting doctoral research. The Graduate School's goal was to assist all graduate programs, faculty and students by providing training in the core curriculum aspects of RCR followed by discipline-specific specialized training delivered by the programs and the students’ research mentor. The program provides a single, uniform RCR training course for all Ph.D. students and postdoctoral scholars at the university that would meet national standards and reduce burdens on doctoral programs who might otherwise need to create their own courses. This course was developed in consultation with internal units and other institutions, as well as using resources available on-line [28–31]. Faculty from science, technology, engineering and mathematics (STEM), social and behavioral science and education (SBSE) and arts and humanities (AH) volunteered to develop the curriculum, using a combination of currently existing departmental materials, new materials based on literature reviews and national workshops, and federal funding agency guidance and requirements.

The course incorporates several best practices in RCR training as well as federal funding agency mandates and integrity training requirements set forth by the HLC accrediting body. These include (a) a hybrid delivery format comprising a mixture of online instruction, faculty presented lectures, interactive discussions of case studies, and an essay; (b) a minimum of 8 hours of face-to-face interaction as required by federal funding agencies; (c) a high degree of faculty involvement in the face-to-face lectures and discussion of the RCR content; and (d) delivery of discipline-specific content by the students' departments, with general RCR training provided by the Graduate School (see S1 File for Fall 2018 syllabus).

First year Ph.D. students were required to take the course to ensure all new students received RCR training early in their research careers. After three semesters, course outcomes were evaluated and the curriculum was revised based on student and faculty feedback. The format remained the same, but greater effort was made to increase participation of faculty from SBSE and AH disciplines in the face-to-face lectures. Case studies were revised to incorporate more examples from SBSE and AH.

Between September 2016 and September 2018, the course was offered five times and completed by 647 doctoral students and postdocs (Table 1). After each course, the Graduate School evaluated outcomes through student surveys administered through Qualtrics online survey tool (see S4 File for survey instrument). The survey was distributed to all students enrolled in the course through an anonymous link sent by email. Students were given three weeks to respond to the survey. Survey responders were given the option to enter into a drawing for one of five $25 gift cards through a separate link. Students were informed the survey would be used to enhance and modify the course and completion of the survey was voluntary. Once the survey closed, responses were analyzed using descriptive statistics to provide a summary of responses. Later, a survey of graduate directors was added to the process. The student surveys were approved by Wayne State University’s Institutional Review Board (IRB#104317B3E).

**Table 1:** Course enrollment by semester.

Semester	Total
Fall 2016	125
Winter 2017	107
Fall 2017	166
Winter 2018	78
Fall 2018	171
**Total **	**647**

The survey was distributed to all 647 students enrolled in the course; 322 students submitted responses of which 278 were fully completed. To be fully completed, the student needed to provide their departmental and or major information. Of the 278 respondents, 262 were Ph.D. students and 16 were postdoctoral scholars. Respondents were from STEM (n = 148), SBSE (n = 95), and AH and additional fields (n = 35) as shown in Table 2. The survey includes the following broad categories of questions: (a) students’ overall experience of the course, including whether they would recommend it to their peers. If students did not recommend the course, they were prompted to include open-ended text comments to contextualize their response; (b) student perceptions of learning in each of the four stages of the course and the extent to which they felt the course or stage was valuable in their development as a scholar; (c) student rankings of the in-person lecture topics to assess perceptions of value to their training and provide information about further development of course material; and (d) student perceived confidence to carry out behaviors consistent with the RCR training. Analyses were conducted by major subfield (STEM, SBSE and AH) to determine if the course was meaningful to students across disciplines.

**Table 2:** Student survey responses by discipline.

Semester	STEM	SBSE	AH	Total
**Fall 2016**	**26**	**17**	**8**	**51**
PhD Students	17	17	8	42
Postdoctoral Scholars	9	0	0	9
**Winter 2017**	**25**	**15**	**3**	**43**
PhD Students	24	14	3	41
Postdoctoral Scholars	1	1	0	2
**Fall 2017**	**35**	**21**	**5**	**61**
PhD Students	35	20	5	60
Postdoctoral Scholars	0	1	0	1
**Winter 2018**	**19**	**21**	**6**	**46**
PhD Students	18	20	6	44
Postdoctoral Scholars	1	1	0	2
**Fall 2018**	**45**	**21**	**13**	**77**
PhD Students	43	21	13	75
Postdoctoral Scholars	2	0	0	2
**Total**	**148**	**95**	**35**	**278**

For stage 1 (CITI Training), stage 2 (in-person workshop), and overall course experience, multiples choice questions were used to assess perceived learning and perceived values. Response options for perceived learning included ‘I learned a great deal’, ‘I learned a little bit’, or ‘I did not learn about good research practices in this course’. Response options for perceived value were ‘extremely valuable’, ‘slightly valuable’ and ‘not at all valuable’. Percentages were calculated based on the responses by disciplines. Respondents were also asked to rate the in-person workshop topics from 1 to 8, with 1 being the most valuable and 8 being the least valuable. For stage III (departmental training), a list of methods of delivery for RCR training at the department level was provided. Respondents were asked to select all that applied. The responses were summarized by count for each discipline. Finally, respondents were asked if they would recommend the course. Those that indicated they would not recommend the course were asked to provide additional comments to explain their response. First, two people coded responses for main themes independently. They then met to review coding and reach consensus where they differed. The main themes were then summarized by count for each discipline.

A survey of graduate directors survey sought to gather information on RCR training at the department level (see S5 File). Respondents were ask to provide their department and school/college information. To gather information on RCR training methods, a question similar to the student survey was included. They were provided a list of training delivery methods and asked to select all that applied. Responses were summarized by count. Respondents were also asked to rate RCR training topics critical to students in their department. A list of nine topics was provided and the respondent were asked to select no more than four. The remaining eight questions were multiple choice with response options of ‘yes’ or ‘no’. They were asked if their research required IRB approval; if they had formal training in RCR; if RCR training was part of their stated learning objectives; if the course should be required for all PhD students, and if they have made changes to RCR training in their departments since the course was implemented. Those that responded yes to this question were asked to indicate if their departments had increased or decreased training. All responses were summarized using percentage of overall responses.

### Limitations

The main limitation of this study is a lack of demographic information. It was the decision of the instructors to make the student surveys anonymous. Questions about race, ethnicity and prior research training were not included on the survey. Although we do know demographics for course enrollment, it is not possible to link this information to survey respondents, therefore it is not provided.

### Ethical approval

This project was conducted with the approval of the Wayne State University Institutional Review Board (IRB#104317B3E). A waiver of written documentation of consent was granted by WSU IRB since 1) risk was no more than minimal; 2) the research involved no procedures for which written consent is normally required outside the research context; 3) the consent process is appropriate; and 4) an information sheet disclosing the required and appropriate additional elements of consent disclosure is provided to participants.

## Results

### Overall course experience

**Fig 1:**
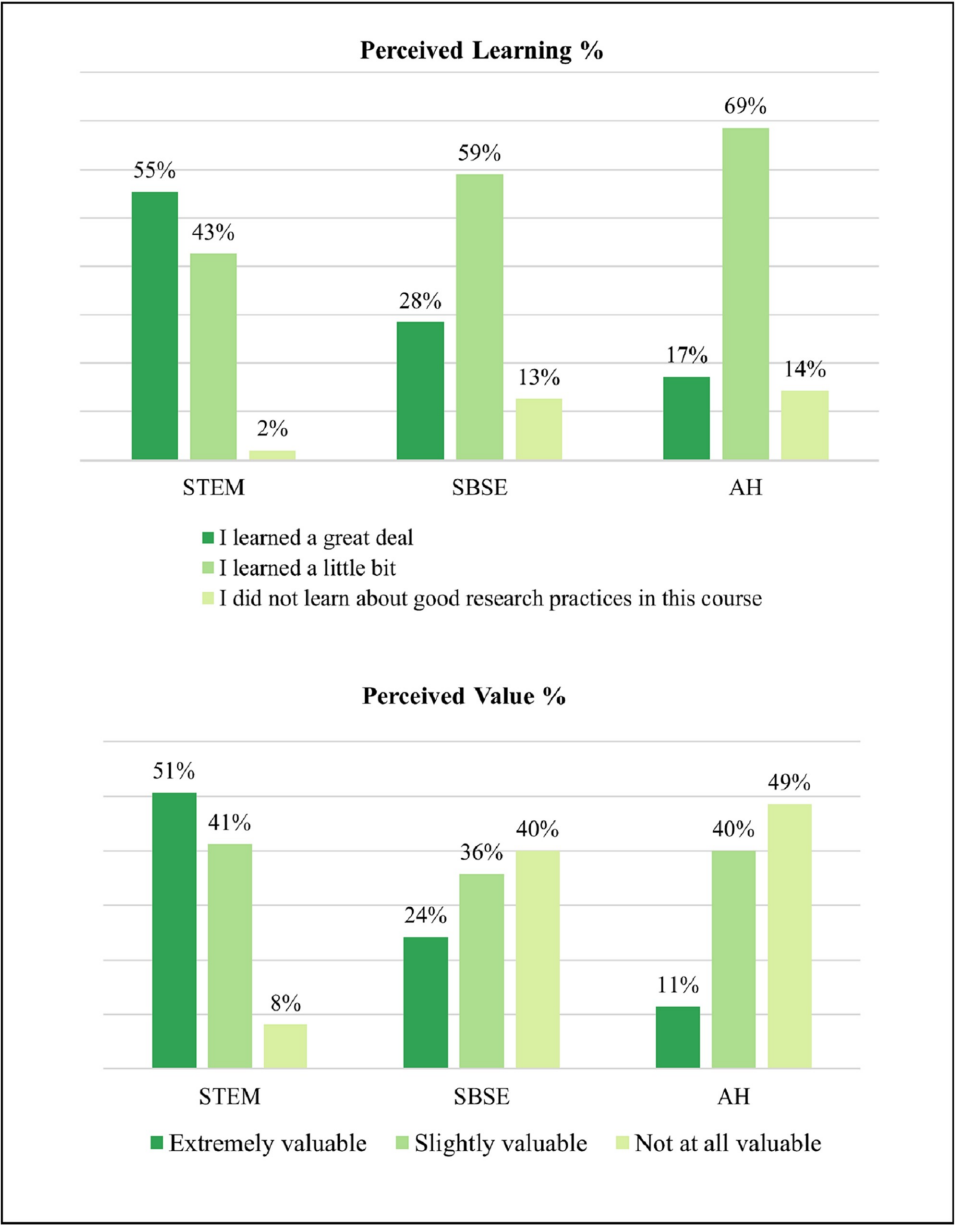
Overall course experience (groups).

The majority (93%) of students reported learning “a little bit” or “a great deal” within the course. However, students’ appraisal of the course’s value differed depending on discipline. Students in STEM fields found the course the most meaningful, followed by SBSE students, with AH students finding the course the least meaningful. The majority of students (62%) said they would recommend this course to other students. Parallel to their perceived learning, endorsement of the class also varied by discipline. The differences by broad disciplinary area are shown in Fig 1.

**Fig 2:**
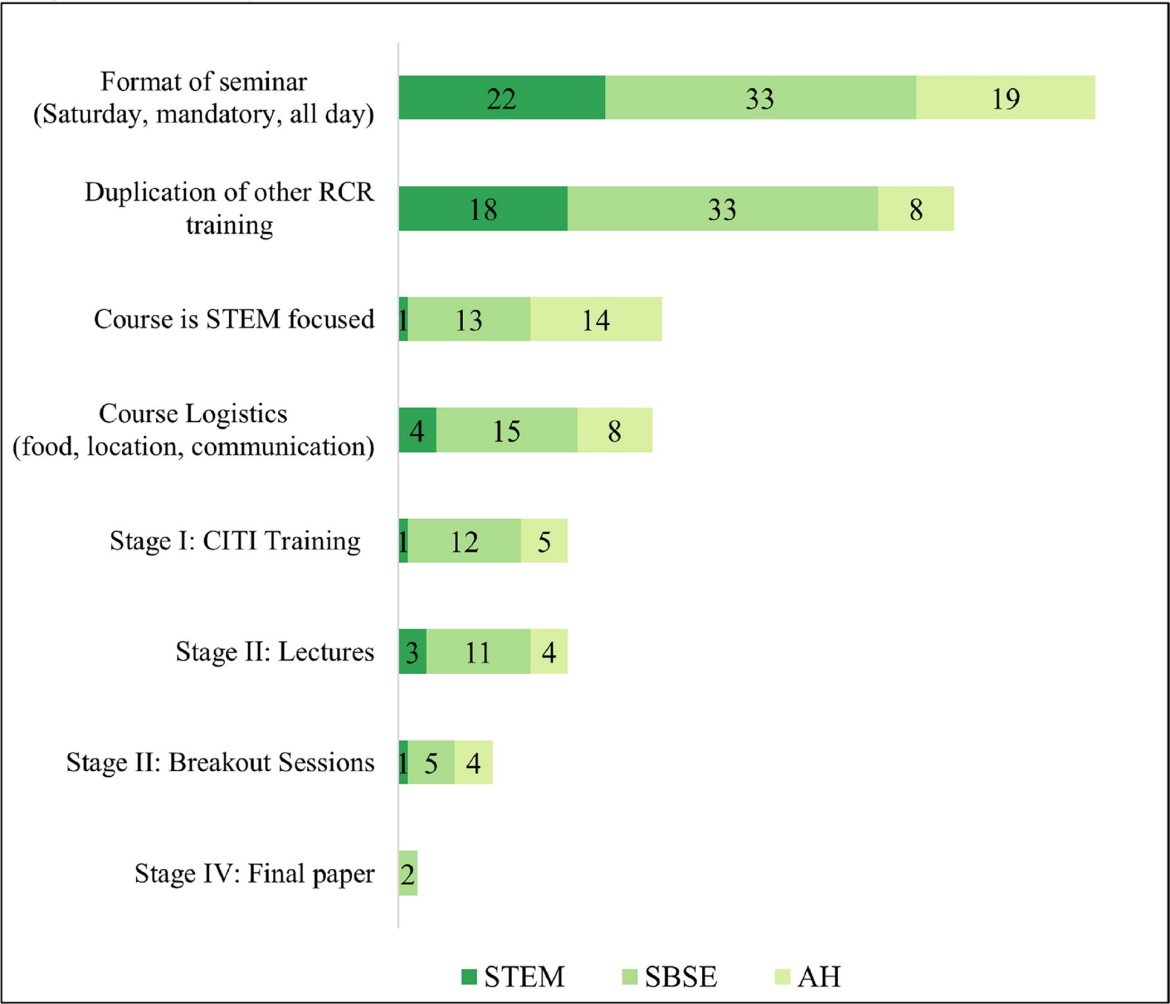
Summary of student comments.

Students who did not recommend the course were asked to provide comments found in Fig 2. The most frequent can be generalized into three categories: (a) some students disliked the format of Stage II, the Saturday all-day in-person workshop. They recommended the all-day format be split into modules, brown-bag talks, or incorporated into their new-student orientation; (b) students felt the course was a duplication of the CITI and/or departmental training. It may be advisable for faculty presenters to take the CITI RCR course a month before the workshop to avoid duplicating CITI content for the Stage II workshop. In some cases, students reported that their undergraduate or pre-doctoral training was sufficient and covered many of the topics within the RCR course; and (c) SBSE and AH students noted that the RCR course addressed topics of little relevance to them (e.g., wet lab procedures, biohazard safety). However, SBSE and AH students acknowledged that the course addressed at least some topics that were relevant to them, mentioning survey design and administration; human subjects research in school settings; and breaking confidentiality to ensure participant safety.

### Evaluation of Stages I, II, and III

**Fig 3:**
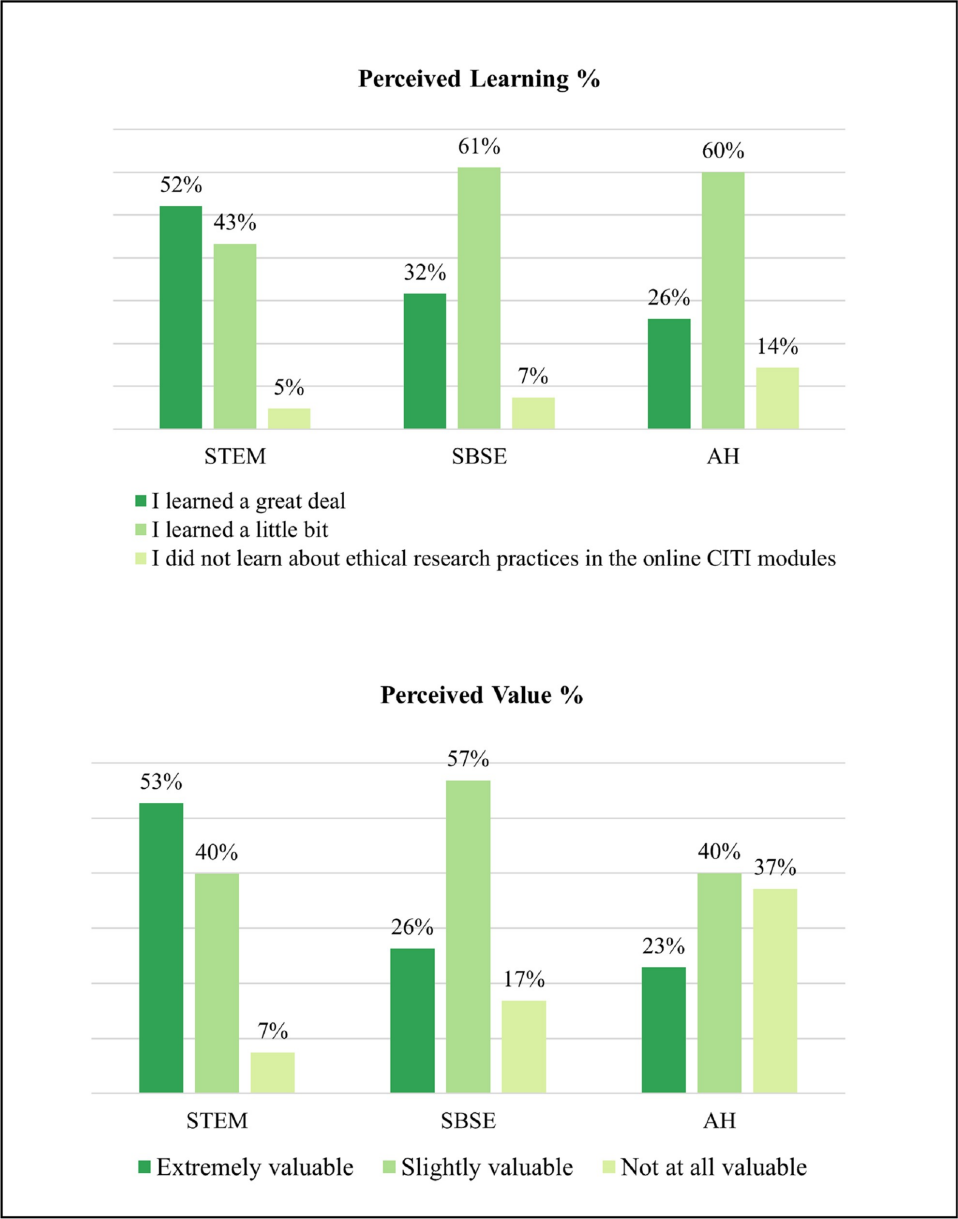
Stage 1: CITI training modules (by discipline).

Across disciplines, the vast majority of students reported high learning of content in Stage I, the online CITI module. Fig 3 shows STEM students were more likely to report they “learned a great deal” from the CITI module while SBSE students were more likely to report they “learned a little bit”. However, many AH students (37%) reported the CITI Training modules were not valuable to their development as scholars. Given these findings, it may be of great value to have CITI training modules designed specifically for SBSE and AH disciplines.

**Fig 4:**
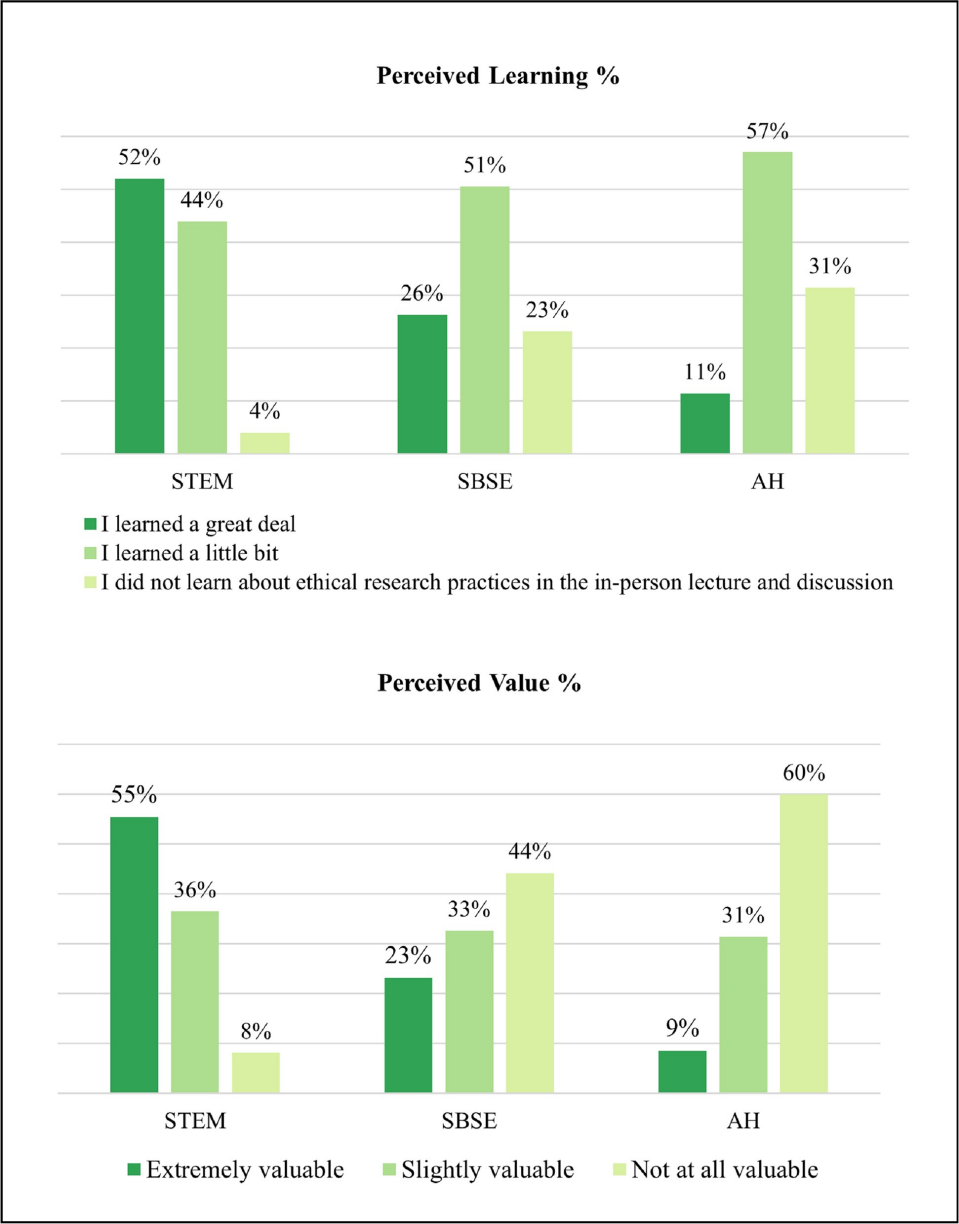
Stage II: In-person lectures and discussion (by discipline).

Stage II ratings revealed that higher proportions of STEM students reporting having “learned a great deal” during the in-person lectures and case study discussions, as compared to the other disciplines as shown in Fig 4. SBSE and AH students were more likely to report the in-person lecture and discussion as “not at all valuable” while STEM students were more likely to report them as “extremely valuable.” Comments regarding the in-person training largely showed that students perceived the lectures and case studies to be most relevant to the STEM disciplines. Participating faculty, including the postdoc discussion leaders, however, were disproportionately representative of the STEM areas, which may have affected the perceived value of the in-person training.

Students also were asked to rank the lecture portion of Stage II from most to least valuable. Table 2 summarizes the number of students by discipline that ranked a lecture as either most valuable or second most valuable. We find general agreement across disciplines of the value of the mentoring and authorship/plagiarism lectures. The peer review lecture was the third highest ranked across all fields, although STEM students found other lectures more valuable. However, for future revisions to the overall course, Table 3 provides helpful guidance about which in-person lecture topics might remain a part of the universal course, and which might be more usefully delivered in broad disciplinary breakout sessions.

**Table 3:** Stage II: Ranking of lectures.

1st or 2nd Choice	STEM	SBSE	AH	Total
Mentoring	63^a^	40 ^a^	19 ^a^	122 ^a^
Conflict Resolution	29	23^c^	3	55
Effective Communication Strategies	26	19	8	53
Data Management and Recordkeeping	37^c^	16	4	57
Reporting Misconduct and Whistleblower Protection	29	16	4	49
Peer Review Process	22	23^c^	14^b^	59^c^
Authorship and Plagiarism	62^b^	33^b^	12^c^	107 ^b^
Conflict of Interest	28	20	6	54

^a^The most valuable lecture

^b^The second most valuable lecture

^c^The third most valuable lecture

**Fig 5:**
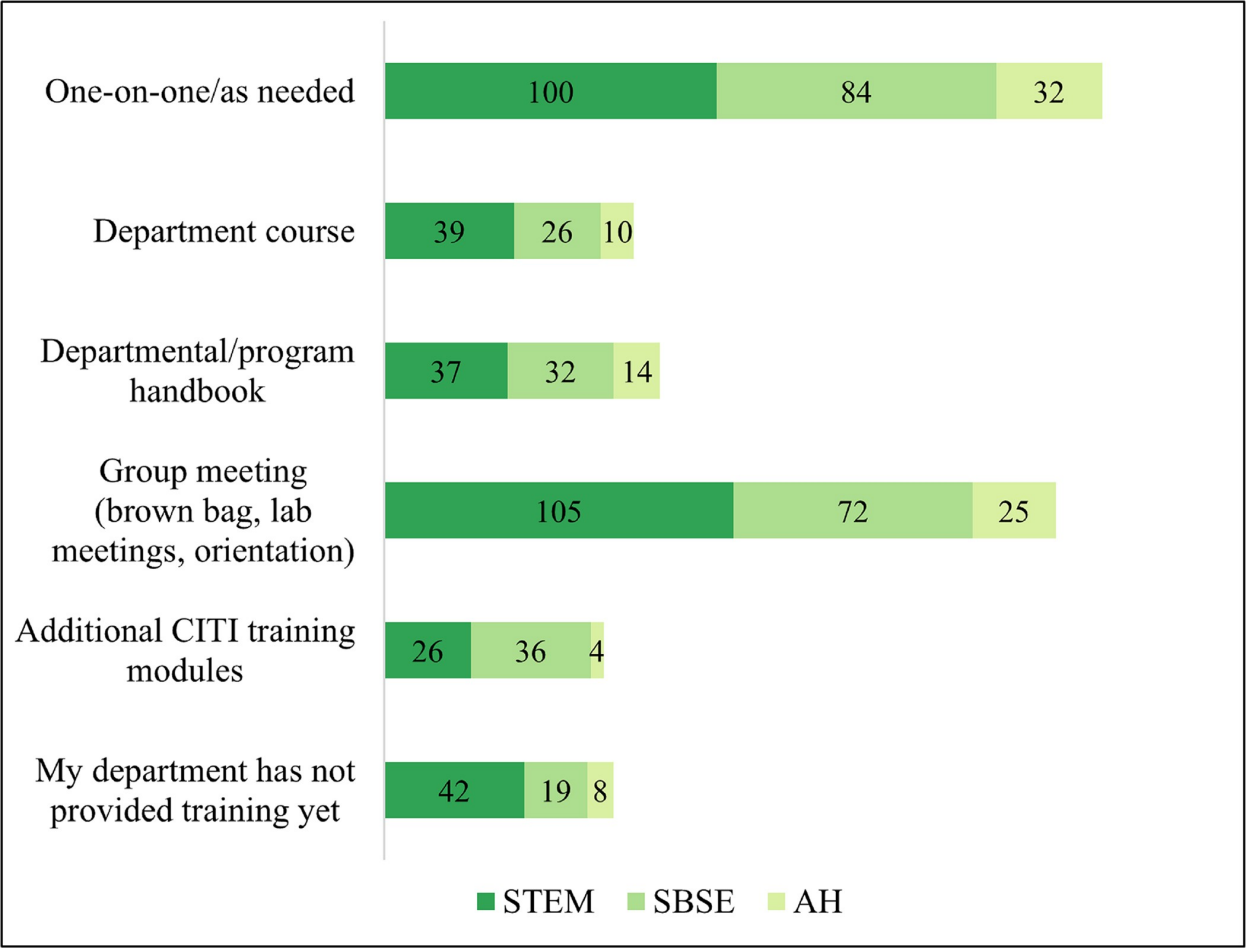
Stage III: Departmental RCR training activities by discipline.

For Stage III, discipline-specific training delivered by department and programs, reflects variability in timing of RCR instruction across programs, but also the fact that building a culture of systematic and intentional RCR instruction takes time. In total, 69 students (42 STEM, 19 SBSE, and 8 AH) had not yet received program-specific training when they filled out the survey difference in how the discipline-specific stage of RCR training is provided (students could select as many options as were utilized by their program shown in Fig 5). STEM students reported receiving RCR training mainly in a group setting, such as their department’s orientation or lab meetings (n = 105); one-on-one consultations (n = 100); and/or part of their course work (n = 39). SBSE students reported receiving RCR training in one-on-one consultations (n = 84), in a group setting, such as their department’s orientation (n = 72), and/or additional CITI training modules (n = 36). AH students reported one-on-one consultations (n = 32), in a group setting, such as their department’s orientation (n = 25), and departmental/program handbooks (n = 14).

We can see that formal discipline-specific training is more likely in STEM, while *ad-hoc* methods are more common in SBSE and AH. The large number of students responding that training took place one-on-one, as-needed, or was provided via a departmental handbook leads us to believe that the Graduate School’s university-wide RCR course fills an important gap for many students, whether or not they immediately appreciate the value of the course.

### Confidence to engage in ethical behaviors

Finally, student respondents were asked to identify perceived barriers to their abilities to engage in ethical behaviors, choosing from a list of responses (respondents could choose more than one barrier). The majority of respondents reported that they did not perceive any barriers (STEM 63%; SBSE 61%, AH 60%). However, a number of students reported potential barriers, including avoiding conflict whenever possible (STEM 13%; SBSE 12%; AH 6%) and fear of retaliation by a colleague or mentor if they were to report suspected research misconduct (STEM 16%; SBSE 15%; AH 14%). A number of students also reported that the desire to give others “the benefit of the doubt” (STEM 15%; SBSE 14%; AH 12%) as a barrier. These responses help inform future course design for Stage II, encouraging us to increase instruction about how to handle situations of conflict or uncertainty, and where to turn to for help within the institution in these types of cases. Future course modifications will identify specific individuals to whom students can turn in these situations such as the department chair or graduate program director.

### Faculty feedback

In an effort to better understand the department contexts of the university-wide RCR training, we conducted a survey of graduate directors to seek their feedback on the course in early 2019. Approximately 70 graduate directors were contacted, yielding 24 responses across a broad spectrum of disciplines. Sixty-eight percent of graduate directors responding said RCR training should be mandatory for all PhD students. The survey further revealed that since course implementation, many departments have not made changes to the RCR related activities offered. However, four respondents indicated that their departments made changes. Three increased RCR training offered to students and one decreased its offerings. The top two methods of delivering RCR training at the departmental level were one-on-one discussions with an advisor/mentor/supervisor or graduate director (27%), and a department class for credit (17%). Research misconduct and plagiarism were cited as the two areas most critical for graduate students. Finally, half of those responding said they do not need IRB approval for their research. Finally, most had been trained in RCR practices earlier in their careers.

## Discussion

The increasing move towards interdisciplinary approaches in research and beyond makes it necessary to train the next generation of PhDs using broader and more varied perspectives. To that end, Wayne State initiated mandatory ethics training for PhD students in all disciplines. This comes as high-profile research misconduct cases continue to fuel national concerns about integrity in research. In our experience, students in varied disciplines appear to have different views on the usefulness and utility of robust training. Further research is needed to understand how to fully engage SBSE and AH disciplines in training.

The goal of the training was two-fold: to inculcate all students with an appreciation and knowledge of research ethics, and to help students understand ethical issues in a diverse set of disciplines. The goal is to better prepare our students for a workforce which increasingly values interdisciplinary teams and research collaborations. We have learned that developing an RCR training course designed to meet the needs of all is an undertaking that requires extensive planning, continuous program improvement, and partnership of all stakeholders. We believe that the implementation of the university-wide course has increased engagement and awareness of RCR across the university, and is creating a culture that values its importance and relevancy.

**Fig 6:**
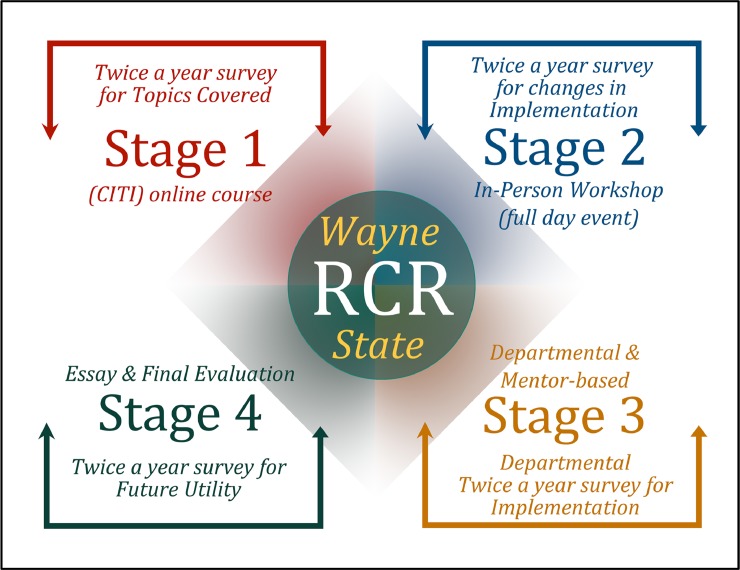
Four stages of the RCR course.

Addressing the specific needs of students in each discipline is still a work in progress. As shown in Fig 6, using an evidence-based approach, the survey data collected from students and graduate directors serves as a tool to continually modify future iterations of the course. This will be a yearly process, which will identify how we can improve the student experience. We have already utilized the information provided in this manuscript to improve course content and layout, event planning for the day-long workshop and the use of discipline-relevant material for workshop breakout sessions.

The feedback we received helped us to enhance course content. Students in SBSE and AH told us that the training had too much of a STEM emphasis. While we attempted to engage a broad range of faculty in course design and implementation, faculty from STEM fields and the biomedical sciences were initially overrepresented. We realized when the course was being created, faculty participation had been solicited from all disciplines, but there were few active participants from SBSE and AH. Since then, we have redoubled our efforts to work with faculty in non-STEM disciplines to increase their participation in course development and implementation. A key component of this process is input in the first stage, which is selection of topics to be covered in an upcoming RCR course and determining the material most pertinent to students.

Based on the student and faculty feedback, we are also changing the full-day workshop. The day will be shorter and students will attend both required and relevant sessions. All students will be required to attend a unified morning session, but afternoon sessions will be more discipline-specific. The workshop will feature frequent breaks to make the workshop more interactive for students. The combination of the workshop and departmental training in Stage III fulfills federal funding agency requirements as there will be a total of eight face-to-face training hours. Students will receive four hours during the workshop and an additional four hours in departmental training.

In conclusion, the very trust of the public depends on the successful adoption of standard ethical training regardless of discipline, at the same time respecting disciplinary differences and needs. Further work is needed to develop set curricula for SBSE and AH RCR curricula to ensure all disciplines have standardized and uniform training, including expanded discipline-specific CITI modules. This would help reduce the disparities between STEM training and the other disciplines. We found that a university-wide RCR program can be the catalyst for an institution-wide engagement with research ethics by engaging a broad group of faculty, gathering continuous feedback and responding to student concerns. One size may not fit all, but one course with careful design and improvement, can meet the needs of students across the university while promoting the importance and value of ethical research practices, especially in the growing landscape of multi- and interdisciplinary research and scholarship.

## Supporting information

S1 File Fall 2018 syllabus. (PDF)

S2 File CITI training. (PDF)

S3 File Sample of RCR case studies. (PDF)

S4 File Student survey. (PDF)

S5 File Graduate directors survey. (PDF)
