# Supplementary material for: Research ethics in inter- and multi-disciplinary teams: Differences in disciplinary interpretations
Source: PLoS One. 2019 Nov 27;14(11):e0225837. doi: 10.1371/journal.pone.0225837 (PMC6881010; doi:10.1371/journal.pone.0225837)
Supplement: S1 File — (PDF) [file pone.0225837.s001.pdf]

Graduate School 0900 (GS0900)
Essential Research Practices: Responsible Conduct of Research
Fall, 2018 – Zero credit hours

Course Coordinator/Instructor:

Dr. Todd Leff — tleff@wayne.edu
Assoc. Professor & Assoc. Dean of the Graduate School — Phone: 313-577-3006
2416 Integrative Biosciences Bldg.

Instructors:

WSU Faculty:

Dr. Philip Cunningham, Professor & Assoc. VP for Research	ad4487@wayne.edu
Dr. Joan Dunbar, Assoc. VP for Technology Commercialization	aj0824@wayne.edu
Dr. Theresa Hastert, Professor, Dept. of Oncology	fs9450@wayne.edu
Dr. Faith Hopp, Professor of Social Work	bb2938@wayne.edu
Dr. Loraleigh Keashly, Professor & Assoc. Dean of College of Fine, Performing & Communication Arts	ad8889@wayne.edu
Dr. Arthur Marotti, Emeritus Professor of English Director of the WSU Emeritus Academy	aa1750@wayne.edu
Dr. Caroline Maun, Assoc. Professor of English	av4495@wayne.edu
Dr. Richard Marback, Professor of English	aa4749@wayen.edu

WSU Postdoctoral Scholars:

Dr. Mariana Angoa-Perez	ay3408@wayne.edu
Dr. Pershang Farshi	fs6022@wayne.edu
Dr. Ambikai Gajan	dv4075@wayne.edu
Dr. Karl Koebke	gr5528@wayne.edu
Dr. Deepthi Yedlapudi	fy4604@wayne.edu

Time/Location:

Date: Saturday, September 29, 2018

Time: 8:00 a.m. – 12:30 p.m. — General Lectures Bld, Room 100, 5045 Anthony Wayne Ave

1:00 – 4:00 p.m. — Manoogian Hall, Rooms – 54, 58, 68, 160, 166, 235, 237, 243, 289, 293 (room assignments will be provided in advance) 906 W. Warren Ave

All course materials are located on the GS0900 Canvas site

Course Objectives and Corresponding Ability-Based Outcomes (ABOs):

This course is required for all PhD students in their first year, and it is suggested for all postdoctoral scholars. Students and postdocs are allowed to take this course in either the Fall or Winter term, all first year PhD students are required to complete the entire course within the first year of your program. Failure to do so will result in a hold being placed on the student's account blocking them from attaining candidacy status.

GS0900 presents both general and directed instruction/discussion on topics essential to performing Responsible Conduct of Research (RCR) practices. Content for the course is delivered in four stages. STAGE 1: Completion of 11 elective modules within the Collaborative Institutional Training Initiative (CITI) RCR Core Topics online course. STAGE 2: A lecture and interactive based workshop to promote training and discussion in core RCR topics. STAGE 3: A Departmental and/or Mentor based RCR instruction in topics specific to the department's and mentor's field of study. Information from all three stages is central for comprehensive student/postdoc RCR training and to the development of the student. STAGE 4: Completion of the summary essay on general and field specific RCR topics. Specific learning objectives for the course and the ABOs they address are as follows.

The students/postdocs will be able to:

- Understand and utilize proper practices in managing and recording their research product. (ABO 1.1).
- Correlate and present research product in a manner consistent with ethical delivery practices (ABOs 1.1, 1.2, 1.3 and 1.5).
- Identify improper ethical practices in research and understand how to initiate awareness of these practices to the mentor, department and university (ABOs 1.2 and 1.5).
- Apply appropriate ethical judgment when conducting research (ABOs 1.3, 1.5, 1.6).
- Initiate and utilize strategies of mentoring that keeps an open course of communication between mentor and student for continued development of the student. (ABOs 1.4 and 1.6).
- Apply ethical practices specific to the field of focus in all current and future conditions (ABO 1.6).

Recommended Textbooks (none required):

The course adheres to the basic principles of RCR instruction. These principles as described in several publications, and distilled nicely in NIH NOT-OD-10-019 (https://grants.nih.gov/grants/guide/notice-files/NOT-OD-10-019.html). The subject matter however is relevant to all humanities and science fields, includes all recommended general focus areas as well as issues relating to public perceptions of research and practical skills such as approaching difficult conversations. For postdoctoral scholars, the context is tailored in relation to the Core Competencies for Postdoctoral Training Requirements as developed by the National Postdoctoral

Association which are not field specific. Electronic content for the course will be provided and available online at the Blackboard Web site for recommended reading material; there is no textbook for this course.

Detailed Schedule for 4 Stages within the Course:

STAGE 1: CITI Training. (**Must be Completed by September 25th, 2018**)

Students must complete and pass (with an 80% competency) all 11 elective modules outlined within the CITI Training "GS0900 RCR Core Topics Course" that is available at the CITI Web site: https://www.citiprogram.org/index.cfm?pageID=14. **DIRECTIONS for completing the GS0900 specific CITI** training modules required for this course are posted on the course Blackboard page under the title "CITI Training Registration and Core Topics Completion Protocol," and this document is placed within the Content folder on the course blackboard page.

Progression to STAGE 2 of this course is contingent on successful completion of EACH of these 11 CITI training modules, which include the topics listed below. *If you are looking at the CITI course online and it is not listed as GS0900 RCR Core Topics course, or you do not see the following 11 modules, you are likely taking the wrong course and will be required to retake your CITI training.* The following 11 CITI modules are required.

Introduction to RCR
Authorship
Collaborative Research
Conflicts of Interest
Data Management
Financial Responsibility
Mentoring
Peer Review
Plagiarism
Research Misconduct
Research Ethics

STAGE 2: Saturday Interactive Workshop (**Workshop will take place on September 29th, 2018**)

Students must arrive on 30 minutes before the workshop and participate in the entire Saturday program. On time attendance is required to receive credit for attending workshop. Verification of completion of the Stage 1 CITI training requirement will be completed by Dr. Stemmler two days prior to the workshop, so you will not need to show verification of your CITI training. If you have not completed your CITI training two days prior to the workshop, you will not receive credit for completing the CITI training in the morning of the workshop, and may not be allowed to attend the Saturday workshop. If this is the case, please speak directly to Dr. Stemmler.

**Time**	**Lecture Topic**
8:00 – 8:15 am	Introduction (*Dr. Arthur Marotti*)
8:15 – 8:20 am	Class Itinerary (*Dr. Todd Leff*)
8:20 – 8:50 am	Mentoring and the IDP (*Dr. Caroline Maun*)
8:50 – 9:20 am	Conflict Resolution (*Dr. Faith Hopp*)
9:20 – 9:50 am	Communication Strategies (*Dr. Loraleigh Keashly*)
9:50 – 10:20 am	Authorship and Plagiarism (*Dr. Richard Marback*)
10:20 – 10:30 am	Break
10:30 - 11:15 am	Reporting Research Misconduct & Whistleblower Protection (*Dr. Phil Cunningham*)<br>Conflict of Interest (*Dr. Phil Cunningham*)
11:15 – 11:45 am	Peer Review Process (*Dr. Theresa Hastert*)
11:45 – 12:15 pm	Authorship, Ownership and Inventorship (*Dr. Joan Dunbar*)
12:15 – 1:00 pm	Break for Lunch (**Lunch will be provided)**
1:00 – 4:00 pm	Break Out Discussion Sessions on Core Topics (*Led by Postdoctoral Scholars*)

STAGE 3: Field Specific RCR Training by Department and Mentor **(Must Complete by December 10th, 2018)**

Progression to the third stage of the course is contingent on successful completion of both Stage 1 and Stage 2 requirements, or through approval by the course director. Each Department is required to provide advanced RCR training to students in their program; training should be specific to the department's field of interest. Departments are responsible for the development and monitoring the content and delivery of Stage III training. It is suitable for Departments to utilize preexisting training materials already in place which they already used for student RCR instruction. The expectation is that this content delivery should be at least 1 hour in duration.

In addition, each mentor is responsible to initiate a formal conversation with their student to further discuss the mentor's expectations of the student and role in their RCR training. Expectation is that this discussion should be on a one on one basis (although it is at the mentor's discursion) and the discussion should last a minimum of 1 hour.

STAGE 4: ESSAY REQUIREMENT for PASS/FAIL GRADE (**Essay submit by December 16th, 2018)**

Once all three stages of the course have been completed, the student/postdoc will be required to complete a formal 2 full page ESSAY (single spaced, 12-point font, 0.75 inch margins) on the topic that summarizes their formal understanding of general and field specific practices in RCR. The essay should include a description of how they plan to utilize and self-monitor RCR practices in their future studies at Wayne State and beyond. Submission of this 2-full page essay should be completed via upload on the Blackboard page specific to the GS0900 course. Once the ESSAY has been evaluated, the student/postdoc will receive a PASS or

FAIL grade for the course, and this grade will be based on all three stages of the course as well as content in the essay.

Attendance:

Attendance is mandatory for Stage 2. Failure to arrive **on time** or to attend stage 2, or to complete any of the three stages, may result in an incomplete grade for the course. Failure to complete the entire course by the candidacy exam will result in a stoppage in the student's ability to attain their candidacy status.

Exemption Program:

Students with Special Circumstances (i.e., **Religious or Cultural Restrictions only**) who will not be able to participate in the Saturday workshop will be allowed to complete an alternate workshop on January 30th (3:00 pm in room 3138 of Eugene Applebaum Building). The alternative workshop will involve viewing the January 20th lectures online, writing a summary of the individual lecture's content, and finally participating in a comprehensive small group discussion involving case studies covering situations that violate RCR principles. Students exempt from the Saturday course must complete their GS0900 CITI training modules by prior to the assigned workshop date, as indicated above, and provide Dr. Stemmler an electronic verification of CITI course completion by this date. Exempt students are required to complete stage 3 training within their college/department/mentor. Finally, exempt students must submit their overall summative essay that covers the three general questions posted in the essay requirement document by the course completion date listed above.

Classroom Behavior:

Students should make every effort at be on time to class. Late attendance to the class, or if the student frequently leaves the lectures or workshop portion of the Saturday workshop during Stage 2, will result in the student being required to retake the workshop. Students should also pay attention during class. The instructors or course director reserves the right to ask students to leave if they are creating a disturbance. Disturbances could result in a requirement to retake the course and disciplinary actions.

Recording and Transcribing Lectures:

The lecture material presented in this course will be distributed to the students for further review via Blackboard.

Assessment:

Assessment of the content delivery will be divided into three parts. Stage 1: assessment of the CITI training content will be the successful completion of the online survey given after each topic. A passing grade of 80% in the test for each topic is required for migration into Stage 2. Stage 2 and 3: following both stages, including workshop and department/mentor discussions, the student will be asked to summarize how they will implement RCR practices in their academic training and beyond in an essay format. Format for the essay

with specific assessment questions will be posted online and a completed Essay must be posted on Blackboard class page by the date assigned above to receive a passing grade in the term.

Special Learning Needs:

University policy - (University Policy located at http://www.bulletins.wayne.edu/fib/fibd.html): “If you feel that you may need an accommodation based on the impact of a disability, please feel free to contact the course coordinator privately to discuss your specific needs. Additionally, the Office of Educational Accessibility Services (EAS) coordinates reasonable accommodations for students with documented disabilities. The Office is located within the 1600 David Adamany Undergraduate Library, phone: 313-577-1851 (Voice) / 577-3365 (TTD).”

Grading policy:

Students who complete all three stages of the course and successfully submit an assessment essay on Blackboard will receive a grade of Satisfactory or “PASS” associated with their transcripts.

In the Event of Course Failure:

Students who do not complete **all three Stages** of the course will be required to complete the missing components in the next subsequent semester, and they will need to take an INCOMPLETE in the course for the current term. Students/Postdocs that PASS the course will receive a certificate of completion for their personal records. If you receive a FAIL grade, you will require to retake the entire course.

Ability Based Outcomes – PhD/Postdoc Participants
The Graduate School
Wayne State University

The purpose of this document is to define a set of concise outcomes for students completing their PhD at Wayne State University regardless of their respective program. The outcomes outlined in this document are ability based outcomes (ABOs). ABOs define what students will be able to do as a result of completing Responsible Conduct of Training associated with completion of their PhD degree at Wayne State University. ABOs do not define the knowledge skills and attitudes that enable students to meet the outcomes. They define what graduates can do as a result of knowledge, skills and attitudes gained through completing RCR training.

The ABOs will be used in conjunction with other content area measures for curricular assessment.

Upon completion of the RCR training, the student will be able to:

1. Demonstrate commitment to a high standard of applying RCR practices.

    1.1 Maintain professional competence by in utilizing proper data management and recordkeeping in their respective field.

    1.2 Possess the attitudes required to identify and apply research ethical practices.

    1.3 Be able to apply moral reasoning in common research practices.

    1.4 Utilize effective strategies related to their mentoring (ex. IDP) to help advance their academic training.

    1.5 Recognize, initiate and advocate change in issues related moral reasoning in resolving conflicts, issues related to peer review (authorship and peer review), and violations of ethical research practices.

    1.6 Apply practices of responsible conduct of research specific to the student's field of training.
