# Supplementary material for: Research ethics in inter- and multi-disciplinary teams: Differences in disciplinary interpretations
Source: PLoS One. 2019 Nov 27;14(11):e0225837. doi: 10.1371/journal.pone.0225837 (PMC6881010; doi:10.1371/journal.pone.0225837)

S2 File

CITI Training Registration and Core Topics Completion Protocol

TLS 07/16/16

The goal of this document is to guide Students/Postdoctoral Scholars through the process of registering for CITI training and then in the completion of the core topic modules essential for migration to the second Stage in the Wayne State University GS0900 – Responsible Conduct of Research training course. For NEW CITI Users: to begin the process, you will need to register for an account on the CITI training web page: https://www.citiprogram.org/

NEW USER REGISTRATION

Click on the <Register> button located in the middle of the login page. This will redirect you to the registration information web page.

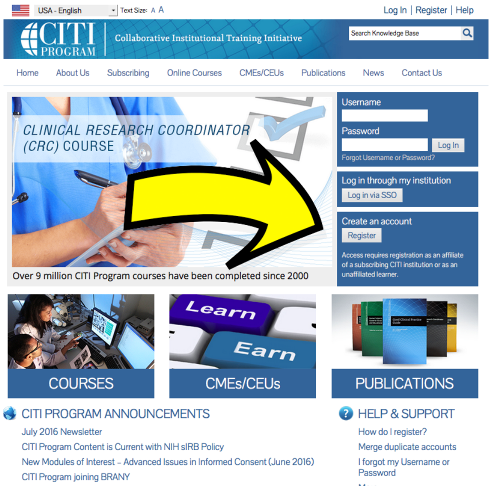

https://www.citiprogram.org/index.cfm?pageID=154&icat=0&ac=0

Step 1: In the "Select your Organization Affiliation" box, type Wayne State University-Detroit, MI. Then click the button indicating <Continue to Step 2>.

Step 2: Type in your full name and your relevant WSU E-mail Address. Click the button to continue to Step 3.

Step3: Create a User Name for yourself (maybe your first initial and full last name). In addition, create and verify a password for your account. *Remember both your username and password for future reference.* Click the button to <Continue to Step 4>.

Step 4: Type in your country of origin. Click the button to <Continue to Step 5>.

Step 5: In response to the Question "Are you interested in the option of receiving Education Unit (CEU) credit for completed CITI Program courses", you **do not need** to select yes, easiest if you hit the NO button. Click the button to <Continue to Step 6>.

Step 6: Complete all the relevant boxes. You are required to complete the boxes with a (*) before the box title. Click the button to <Continue to Step 7>.

<u>Step 7:</u> This step allows you to define the courses that are applicable to your position and your program requirements. You can activate other courses at another date, however for the GS0900 course you need to select under Question 4 the "GS0900 RCR Core Topics Course". For Question 8, DO NOT SELECT any designation, LEAVE THIS SECTION BLANK. If you select, you will have a duplicate set of courses to take and this is not necessary. Finally, click the <Complete Registration> button.

You will now be directed to a web page that will inform you that "Your registration with Wayne State University-Detroit, MI in complete. Hit the <Finalize registration> button to continue. You wil now be directed to the course page: https://www.citiprogram.org/members/index.cfm?pageID=50&submitSuccessful=1

Completion of the GS0900 RCR Core Topics Course

You are now on the CITI web page that will allow you to access each of the 11 Core Topics modulesthat you are required to complete at an 80%grade or higher to progress to STAGE 2 in the GS0900 RCR Required course.

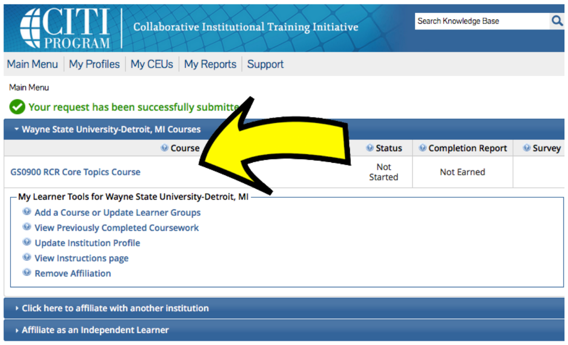

Click on the blue text indication "GS0900 RCR Core Topics Course" to begin. Note: there is a section of blue text in the My Learner Tools section where you can click and add courses in the future.

You will now be redirected to the individual Core Topics Course page. It will provide you with a current score as well as an individual score for each core module (as well as listing if you completed each module). Before you can start completion of each Core module, you will need to click on and complete the "Complete the Integrity Assurance Statement before beginning the course" text. Once you agree with the Integrity Assurance Statement, you will be redirected back to the page listing each of the course modules.

Since you have just begun the course, each of the course modules will be listed as incomplete. The first of the modules "Introduction to RCR (RCR Basics)- New (ID:17009)" will be activated in blue. Click on the text for this required module to begin delivery of the content specific to this module.

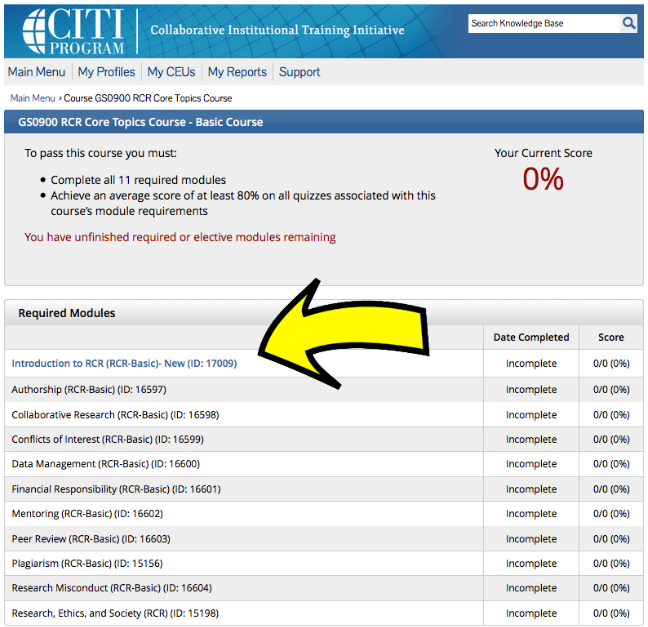

At the bottom of the Introduction section web page will be blue text indicating "Take the quiz for Introduction to RCR (RCR-Basic)- New" which will allow you to take the quiz for this section. If you score well on the quiz (i.e., above 80%), you can select the blue option at the bottom of the page which will allow you to "Take the next required module". If you did not do well, you will be given the option to "View this module again and retake the quiz".

If you "Return to the module list for this course", you will see the copurse that you have completed and their relevant score. In addition, the next module will be highlighted in blue, so you can click on that text to activate the content delivery for the next module.

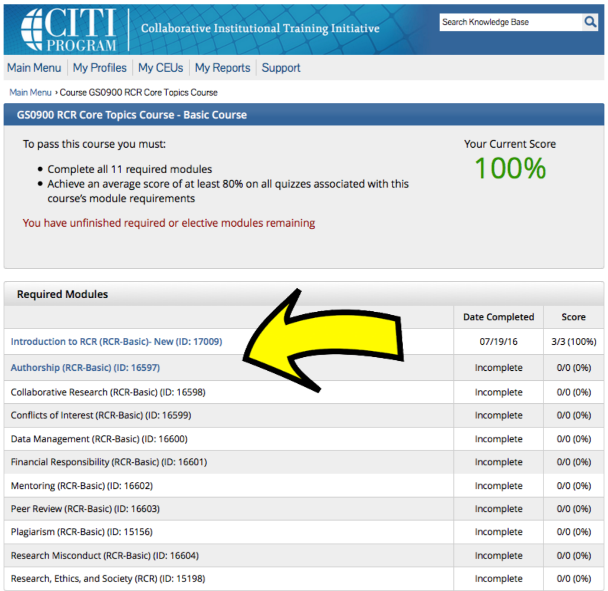

In the same process, you will be able to select, review and take the quiz for all 11 of the required modules for the CITI portion of the GS0900 course. Once all modules are completed, and your total current score is over 80%, then print a copy (or take a picture) of the completed grading page. **Bring this printout with you to the GS0900 STAGE 2 workshop for verification. You will need this to attend the lectures and the breakout sections associated with the day long workshop.**

CURRENT REGISTERED CITI TRAINING USERS

If you already have a CITI account, either from WSU or a different University, then log in to the CITI Web page: https://www.citiprogram.org/

S2 File

You should be directed to the course modules page. From here you have access to menus that allow you to "Update Institutional Profile" and "Add a Course or Update Learner Groups". If your affiliation is not Wayne State University, change it so that it is. Then you can Add the GS0900 Core Topics Course to your course listing by following STEP 7 listed on the first page of this protocol.

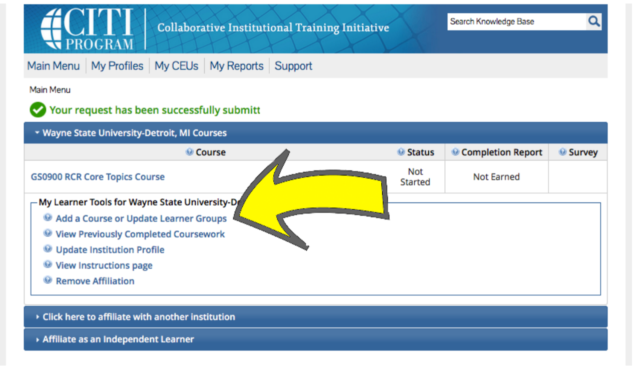

Supplement: S2 File — (PDF) [file pone.0225837.s002.pdf]
