# Supplementary material for: Research ethics in inter- and multi-disciplinary teams: Differences in disciplinary interpretations
Source: PLoS One. 2019 Nov 27;14(11):e0225837. doi: 10.1371/journal.pone.0225837 (PMC6881010; doi:10.1371/journal.pone.0225837)
Supplement: S3 File — (PDF) [file pone.0225837.s003.pdf]

S3 File

RCR Case Study Discussions

Afternoon Breakout Session Materials

Case 1: Data Ownership

Robert Wright is a graduate student who was chosen for a university grant-funded service assignment where he assists with archival research and drafting components of a large editing project. The Principle Investigator, Professor Janson, won several grants (both internal to the university and from external agencies) and took reference photographs of archival materials which include manuscripts of an author who is now little known but whom she hopes to bring to the academic community's and public's attention with the edition of an unpublished novel from manuscripts. Robert's tasks over the year of the appointment involve reviewing the reference photographs, cataloging them in a database and drafting endnotes that refer a reader to the locations of these manuscripts when necessary. Professor Janson reviews all of his work, makes final decisions about which variations in the text will be included, and edits and expands the notes that Robert drafts to include information specific to the editorial decisions when applicable. They work on this project for the year and during the time he is working, Robert becomes interested in this author and wishes to do his own work on the subject. At the conclusion of his work on the project, Robert asks for continuing access to the files of the edition while it is under review with. Additionally, he asks to see the Introduction that Professor Janson is completing. Professor Janson does not share the Introduction with him and asks him to refrain from publishing his work on this author until the edition has had the opportunity to go through peer review and appear in its final form. Over the next year, Robert proceeds to deliver a conference paper that references the as-yet unpublished edition and completes an article he places under review with a peer-reviewed journal.

Questions for essay discussion:

1. Who owns the research data (in this case the reference images of manuscripts and the files that include the edition and notes)?

2. Does the Professor/PI have the right to ask that the graduate student refrain from presenting or publishing from the unpublished work?

3. What could have been done to better define the boundaries Robert should expect for using the archival material in his own work?

4. Are the policies of how you may use data in your own work clearly defined in research projects in which you are engaged?

S3 File

RCR Case Study Discussions

Afternoon Breakout Session Materials

Case 2: Intellectual Property

Justine, a graduate student and Graduate Teaching Assistant, is in the department's mailroom to make photocopies of her final exam for her composition students near the end of the semester. She runs into Professor Unger, and they agree it was a very good semester in the seminar on Virginia Woolf, which just concluded. Professor Unger is a leader in the field of transnational modernist literature and the current president of a regional branch of the main national professional organization in their field. He compliments the paper Justine completed a few days ago; in fact, he was about to put the graded copy in her mailbox. He asks her if he could have a copy for his files. Flattered, and assuming he collects strong papers to refer to in his future teaching, Justine says yes. Professor Unger copies the paper, gives her the original, and they wish each other a good end to the semester.

Several years later Justine has defended her dissertation, accepted her first position as an assistant professor, and developed her seminar paper on Virginia Woolf into an article that was recently published by a peer-reviewed journal that focuses on the author. One day she receives the quarterly journal from the regional professional organization and notices that Professor Unger has published a paper on Woolf on a topic very similar to the one she pursued in her seminar paper. She reads the article and finds that he has duplicated (and expanded) her argument in his own words and has used the textual examples she had in her own paper in the same order in his article.

Questions for discussion:

1. Is Professor Unger's use of Justine's work plagiarism?
2. Who could Justine speak with if she wanted to pursue the matter?
3. How can situations like these be prevented?
4. How should this situation be resolved?

Case Study Developed by the Faculty at Wayne State University (2017)
