# Supplementary material for: Research ethics in inter- and multi-disciplinary teams: Differences in disciplinary interpretations
Source: PLoS One. 2019 Nov 27;14(11):e0225837. doi: 10.1371/journal.pone.0225837 (PMC6881010; doi:10.1371/journal.pone.0225837)
Supplement: S4 File — (PDF) [file pone.0225837.s004.pdf]

Student RCR Survey

Q1 Overall, how much did you learn about good research practices in GS 0900: Essential Research Practices?

- ○ I learned a great deal
- ○ I learned a little bit
- ○ I did not learn about good research practices in this course

Q2 Overall, to what extent was this course valuable to you in your development as a scholar/researcher?

- ○ Extremely valuable
- ○ Slightly valuable
- ○ Not at all valuable

Q3 How much did you learn about ethical research practices in the online CITI module?

- ○ I learned a great deal
- ○ I learned a little bit
- ○ I did not learn about ethical research practices in the online module

Q4 To what extent was the online CITI module valuable to you in your development as a scholar/researcher?

- ○ Extremely valuable
- ○ Slightly valuable
- ○ Not at all valuable

Q5 How much did you learn about ethical research practices in the in-person lecture and discussion?

- ○ I learned a great deal
- ○ I learned a little bit
- ○ I did not learn about ethical research practices in the in-person lecture and discussion

Q6 To what extent was the in-person lecture and discussion module valuable to you in your development as a scholar/researcher?

- ○ Extremely valuable
- ○ Slightly valuable
- ○ Not at all valuable

Q7 Please drag and drop the choices below to rank order the in-person module topics. A ranking of 1 indicates the module that was most valuable to you, a ranking of 2 indicates the ranking that was second in value, and so on through a ranking of 8, which indicates the least valuable module.

- ◯ Mentoring
- ◯ Conflict Resolution
- ◯ Effective Communication Strategies
- ◯ Data Management and Recordkeeping
- ◯ Reporting Misconduct and Whistleblower Protection
- ◯ Peer Review Process
- ◯ Authorship and Plagiarism
- ◯ Conflict of Interest

Q8 What format(s) have your department or program used to provide you with additional training in ethical research practices beyond GS 0900 (Select all that apply)?

- ◯ My department has not provided me with additional training yet
- ◯ Departmental/program orientation
- ◯ Departmental/program handbook
- ◯ Department course for credit
- ◯ Department course for no credit (0-credit course)
- ◯ Brown bag series
- ◯ Additional CITI training modules
- ◯ Lab meeting discussions
- ◯ One-on-one consultations with your advisor/mentor/supervisor/graduate director
- ◯ As needed discussions with faculty
- ◯ Other ____

Q9 How much did you learn about ethical research practices in your department or program training?

- ◯ I learned a great deal
- ◯ I learned a little bit
- ◯ I did not learn about ethical research practices in my department

Q10 To what extent was your department or program training in responsible conduct of research practices valuable to you in your development as a scholar/researcher?

- ◯ Extremely valuable
- ◯ Slightly valuable
- ◯ Not at all valuable

Student RCR Survey

Q11 After taking GS 0900, do you believe you have **acquired knowledge** in the following domains AND do you **feel confident** in carrying out behaviors in each domain?

	Did you acquire knowledge?			Do you feel confident in carrying out these behaviors?		
	Yes, a great deal	Yes, a little	No, not at all	I feel very confident	I feel somewhat confident	I don't feel confident
Understand and utilize proper practices in managing and recording their research product						
Correlate and present research product in a manner consistent with ethical delivery practices						
Identify improper ethical practices in research and understand how to initiate awareness of these practices to the mentor, department and university						
Apply appropriate ethical judgment when conducting research						
Initiate and utilize strategies of mentoring that keeps an open course of communication between mentor and student for continued development of the student						
Apply ethical practices specific to the field of focus in all current and future conditions						

Q12 What is the most significant barrier to being able to apply what you learned from GS 0900? (choose all that apply)

- ○ I do not perceive any barriers at this time
- ○ I do not feel comfortable speaking to my mentor about my career path
- ○ I would fear retaliation by a colleague/mentor for reporting suspected research misconduct
- ○ I do not fully understand what data fabrication and falsification are
- ○ I do not fully understand what counts as plagiarism
- ○ I avoid conflict at all costs
- ○ I like to give people the benefit of the doubt
- ○ Other ____

Student RCR Survey

Q13 Would you recommend this course (GS 0900) to other doctoral students and postdoctoral scholars?

- ◯ Yes
- ◯ No

Q14 If you would not recommend this course, please briefly explain why:

Q15 **Demographic Information**

Q16 Sex

- ◯ Male
- ◯ Female
- ◯ Other

Q17 Age ____

Q18 Status

- ◯ Ph.D. Student
- ◯ Postdoctoral Scholar/Fellow

Q19 Department Name ____

Q20 Semester in which you took GS 0900

- ◯ Fall Semester 2018
- ◯ Winter Semester 2018
- ◯ Fall Semester 2017
- ◯ Winter Semester 2017
- ◯ Fall Semester 2016

Q21 Discipline Category

- ◯ Science, Technology, Engineering, and Mathematics (STEM)
- ◯ Social and Behavioral Sciences (e.g., Psychology, Sociology, Anthropology, Political Science, Economics, Business, Education)
- ◯ Arts and Humanities (e.g. English, History, Art and Art History, Communication)
- ◯ Other ____
