# Supplementary material for: Research ethics in inter- and multi-disciplinary teams: Differences in disciplinary interpretations
Source: PLoS One. 2019 Nov 27;14(11):e0225837. doi: 10.1371/journal.pone.0225837 (PMC6881010; doi:10.1371/journal.pone.0225837)
Supplement: S5 File — (PDF) [file pone.0225837.s005.pdf]

Grad Directors RCR Survey

Responsible Conduct of Research - Survey of Graduate Directors

The WSU Graduate School developed a centralized Responsible Conduct of Research (RCR) course (GS- 0900) mandatory for all new Ph.D. students and postdoctoral scholars beginning Fall 2016. The course is designed to assist all graduate programs by providing centralized training in the core curriculum aspects of RCR in compliance with the requirements of federal funding agencies.

The course has four components: the online CITI training; a one-day, in-person Saturday workshop incorporating lectures and small group discussions; discipline-specific training delivered by the doctoral program and the trainee's research mentor; and a reflective essay in which students sum up what they have learned. Now that the course has run for two years, we are gathering data to assess and improve our RCR training so that we can best serve all departments, faculty, students and postdocs.

Please take a few moments to help us by filling out this brief survey.
Q1What is your department? ____

Q2 What is your school/college?
- College of Education
- College of Engineering
- College of Fine, Performing & Communication Arts
- College of Liberal Arts and Sciences
- Eugene Applebaum College of Pharmacy and Health Sciences
- Graduate School
- Mike Ilitch School of Business
- School of Medicine
- College of Nursing
- School of Social Work

Q3 How does your department currently provide RCR training? Check all that apply
- RCR training is provided through a department or program orientation
- RCR training is provided through a department handbook
- RCR training is provided through a department course for credit
- RCR training is provided during a department course for NO credit
- RCR training is provided during a portion of a department course for credit
- RCR training is provided at a brown bag or workshop series
- RCR training is provided with additional CITI training modules
- Lab meeting discussions
- One-on-one discussions with the advisor/mentor/supervisor/graduate director
- Other ____
- The Department does not provide RCR training to graduate students

Q4 Has your department changed the way it delivers RCR training since GS-9000 was made mandatory for all PhD students and postdoctoral scholars in 2016?
- Yes (Got to Q5)
- No (Skip to Q6)

Grad Directors RCR Survey

Q5 Please select the way in which you changed departmental RCR training after GS-9000 was made mandatory in 2016

- We have increased our departmental RCR training
- We have decreased our departmental RCR training
- Other ____

Q6 What areas of RCR training are most critical for graduate students in your department? Please select only 4 and rank in order from 1-4.

- Collaborative research
- Conflict of interest
- Data management and data ownership
- Financial responsibility
- Mentoring
- Peer review
- Plagiarism
- Research misconduct
- Research ethics
- Other

Q7 Does your own research require IRB approval?

- Yes
- No

Q8 Did you have formal training in RCR?

- Yes
- No

Q9 Is RCR part of your department's stated learning objectives in Compliance Assist?

- Yes
- No

Q10 Do you think GS-0900 should be required for all PhD students in all disciplines?

- Yes
- No

Q11 Faculty and staff are encouraged to participate in GS-0900 as an instructor or observer. Would you like more information about the course in the future?

- Yes (Please provide access I.D.) ____
- No
